# Intraocular Pressure Variations in Postural Changes: Comparison between Obese and Non-Obese Controls

**DOI:** 10.3390/jcm12185883

**Published:** 2023-09-10

**Authors:** Maddalena De Bernardo, Vincenzo Pilone, Ilenia Di Paola, Ferdinando Cione, Giovanni Cembalo, Pietro Calabrese, Nicola Rosa

**Affiliations:** 1 Eye Unit, Department of Medicine, Surgery and Dentistry, Scuola Medica Salernitana, University of Salerno, 84084 Salerno, Italy; mdebernardo@unisa.it (M.D.B.); nrosa@unisa.it (N.R.); 2 Unit of General, Emergency and Bariatric Surgery, Department of Medicine, Surgery and Dentistry, Scuola Medica Salernitana, University of Salerno, 84084 Salerno, Italy; vpilone@unisa.it (V.P.);

**Keywords:** IOP, ΔIOP, obese patients, non-obese controls, supine position

## Abstract

Background: Comparing intraocular pressure (IOP) changes (ΔIOP) between obese subjects and non-obese controls in relation to different positions: standing, sitting, supine. Methods: the IOP was measured in both obese patients and non-obese controls groups with Tono-Pen AVIA in different positions following this sequence: after 5 min (5′) in the standing position, sitting, supine, supine after 5 min (supine 5′) and immediately after standing. ΔIOP values obtained comparing all positions were, therefore, evaluated. Results: 92 eyes of 46 obese subjects aged between 18 and 59 years (mean 38.07 ± 11.51 years) and of a Body Mass Index (BMI) between 31.84 and 60.65 (mean 41.84 ± 7.05) were evaluated. A total of 48 eyes of 24 non-obese controls aged between 23 and 55 (mean 35.21 ± 11.96 years) and of a BMI between 18.20 and 26.79 (mean 21.04 ± 2.36) were also recruited. In obese subjects, there were statistically significant differences between the IOP in the supine position and the supine positions 5′ with all other IOP measurements (*p* < 0.05). There were statistically significant differences between ΔIOP in both supine positions and prolonged standing positions obtained by obese subjects and non-obese controls (*p* < 0.05). Conclusions: In obese subjects, there is a statistically significant increase in IOP in the supine positions that is significantly greater than the non-obese population. BMI is weakly correlated with IOP and ΔIOP in postural changes.

## 1. Introduction

Glaucoma is a leading cause of blindness worldwide, resulting from optic nerve damage and visual field defects, and eventually inducing visual blindness. It is estimated that it will affect 111.8 million people around the world by 2040 [1]. The elevation of intraocular pressure (IOP) is recognized as the most well-known risk factor related to the glaucoma pathogenesis: it is responsible for both the development and progression of the disease. According to this, to prevent blindness caused by a high IOP, the early diagnosis of glaucoma and proper treatment are needed [2].

Despite progress in understanding the pathophysiology of glaucoma, and the difficulty in obtaining precise measurements in some patients [3,4], the IOP remains the only factor that can be modified by therapy [5,6,7]. In addition to IOP elevation, many other risk factors may influence the development and progression of glaucoma: among these, diabetes, metabolic syndrome and obstructive sleep apnea syndrome (OSAS) [8,9,10]. Some of these clinical conditions are commonly found in obese subjects [11]. Obesity is a chronic systemic condition characterized by a weight excess of greater than 20–25%, compared to the ideal weight for the statural age, which is more commonly expressed with the Body Mass Index (BMI). The World Health Organization defines overweight and obesity as an abnormal or excessive accumulation of fat that poses a health risk. Individuals with BMI between 25 kg/m^2^ and 29.9 kg/m^2^ are considered overweight subjects and individuals with a BMI greater than 30 kg/m^2^ are considered obese [12]. A correlation between obesity and diabetic retinopathy, age-related maculopathy, cataract, and glaucoma has been found in several studies [13,14,15,16,17].

The aim of this study was to compare IOP changes (ΔIOP) between obese subjects and non-obese controls in relation to postural changes.

## 2. Methods

Ninety-two eyes of forty-six obese subjects (14 males) aging between 18 and 59 years (mean 38.07 ± 11.51) and with BMIs between 31.84 and 60.65 (mean 41.84 ± 7.05) were recruited in the Bariatric Surgery department of the AOU San Giovanni di Dio e Ruggi d’Aragona and were included in this prospective, comparative study. In addition, forty-eight eyes of twenty-four non-obese controls (5 males) aged between 23 and 55 (mean 35.21 ± 11.96 years) and with BMIs between 18.20 and 26.79 (mean 21.04 ± 2.36) were evaluated. Participants with a certain diagnosis of glaucoma or with narrow angle were excluded. Also, patients with current or recent use of steroids, or with any ongoing eye disease or with an history ocular trauma or ocular surgery, as well as any condition that affects accurate IOP measurement [18], were excluded from the study.

The study was conducted in accordance with the Helsinki declaration of the World Medical Association. Institutional Review Board (IRB) approval (approving institution: Cometico Campania) and written informed consent from each patient included in the study were obtained. 

All subjects underwent an ophthalmological examination, including IOP measurements, between 9 a.m. and 1 p.m., which were performed by a single observer using a Tono-Pen AVIA (TPA-Reichert Inc., Depew, New York, NY, USA). Only statistical confidence indicator values over 95 were accepted, and with lower values, the measurement was repeated. The Tono-Pen AVIA does not need daily calibration, and the device was calibrated once before starting the trail [17].

Before the measurements, the eyes were anesthetized with oxybuprocaine eye drops. Measurements were obtained in different positions assumed by the patients and controls with this constant sequence: standing after 5 min (5′), sitting, supine, supine after 5 min (supine 5′), and immediately standing again. Laying on one side, stooping over or bending down were not permitted. Both eyes were evaluated.

Descriptive statistics were performed using Microsoft Excel software 2021 (Microsoft 365) (Microsoft Corporation, Redmond, WA, USA). Statistical analyses were performed with SPSS software (Version 26.0 SPSS Inc., Chicago, IL, USA). The normality of data was examined using the exact Kolmogorov–Smirnov test. For pair-wise comparison between IOP measurement in patients, a nonparametric Friedman’s test with Bonferroni correction was used. For pair-wise comparisons between IOP measurements in controls, a repeated-measures ANOVA test with a Bonferroni correction was used. For pair-wise comparisons between ΔIOP of the patients and controls in each position, an exact 1-tailed Mann–Whitney U test was performed. A *p* value < 0.050 was considered statistically significant. Correlations between IOP measurements and biometric parameters were evaluated using Spearman’s rank correlation coefficient (ρ).

The required sample size was calculated with G*Power software (Version 3.1.9.6, Faul, Erdfelder, Lang, & Buchner, 2020. Available at https://www.gpower.hhu.de, accessed on 1 August 2023). Given a Partial η2 of 0.240, calculated by following the example of other studies [18]—a non-sphericity correction ε of 0.820 corrected with the Greenhouse–Geisser method, both calculated with SPSS software, and an Effect Size of 0.562—it was estimated that with a significance level of 1% and a test power of 95%, a sample size of 30 eyes would be necessary.

## 3. Results

Ninety-two eyes of 46 obese subjects, age range 18–59 (38.07 ± 11.51) with a BMI between 31.84 and 60.65 (41.84 ± 7.05) and forty-eight eyes of 24 non-obese controls, age range 23–55 (35.21 ± 11.96), with a BMI between 18.20 and 26.79 (21.04 ± 2.36), were recruited (Table 1).

**Figure 1:**
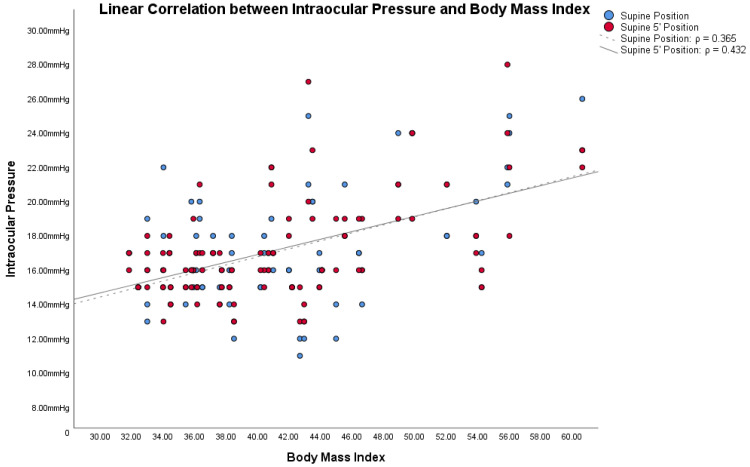
Linear correlations with Spearman’s rank correlation coefficient (ρ) between intraocular pressure (IOP) and Body Mass Index (BMI) in obese subjects.

**Figure 2:**
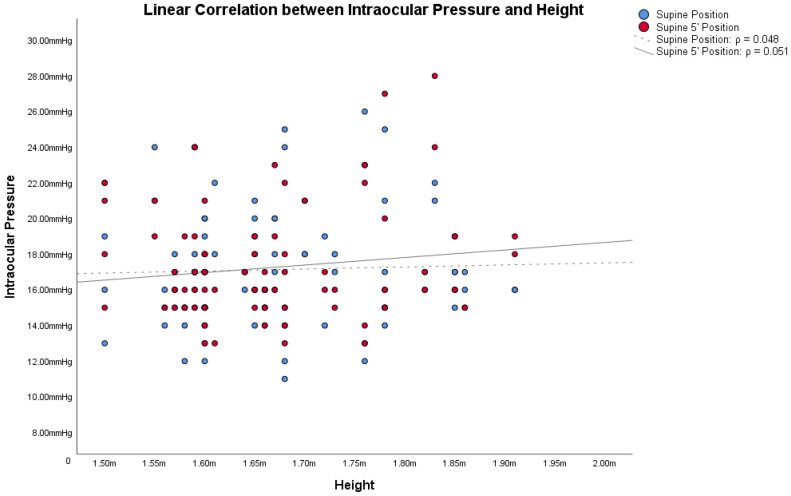
Linear correlations with Spearman’s rank correlation coefficient (ρ) between intraocular pressure (IOP) and height in obese subjects.

**Figure 3:**
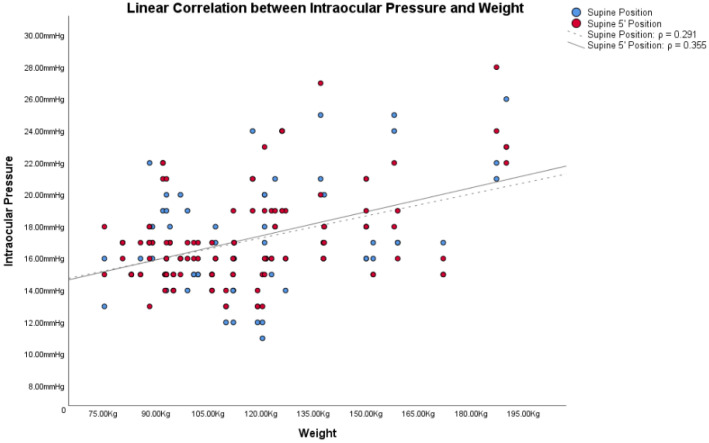
Linear correlations with Spearman’s rank correlation coefficient (ρ) between intraocular pressure (IOP) and weight in obese subjects.

In obese subjects, the Bonferroni post hoc analysis showed statistically significant differences between both the IOP in the supine position/IOP in the supine position 5′, and the other IOP measurement (all *p* < 0.001). Figure 1, Figure 2 and Figure 3 show the correlations between the IOP in the supine positions with the BMI, height and weight; Table 2 reports all the ρ coefficients.

In non-obese controls, the Bonferroni post hoc analysis showed statistically significant differences between the IOP in the supine position 5′, the IOP in the standing position 5′ (*p* = 0.015), and the IOP in the sitting position/IOP in the immediately standing position (both *p* < 0.001); statistically significant differences between the IOP in the supine position and IOP in the sitting position/IOP in the immediately standing position were also detected (both *p* < 0.001). No statistically significant differences between the IOP in the supine and in the standing position 5′ were found (*p* = 0.082).

In the light of these results, only ΔIOP between the supine positions and other positions were compared with BMI, height, and weight, because only in the supine positions were there significant changes. Correlations between ΔIOPs and BMI, height and weight in obese subjects are shown in Table 2. 

**Figure 4:**
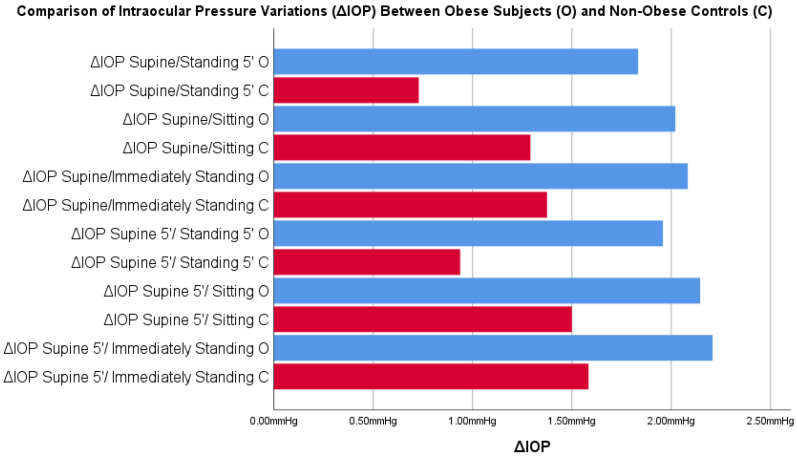
Comparisons between intraocular pressure variations (ΔIOP) in postural changes obtained in obese subjects and in non-obese controls. Blue = supine position; Red = supine 5’ position; (Legend is in the figures).

Comparisons between ΔIOP both in obese subjects and in non-obese controls are summarized in Table 3 and Figure 4. Comparisons between ΔIOP according to gender are reported in Supplementary Tables S1 and S2.

The comparison of ΔIOP Supine/Standing 5′ and ΔIOP Supine after 5 min (supine 5′)/Standing 5′ showed a statistically significant difference between the obese subjects and non-obese controls.

## 4. Discussion

The increase in IOP in the supine position has been previously described in several reports [19,20]. De Bernardo et al. [19] measured the IOP with a rebound tonometer, the Icare PRO (Icare Finland Oy, Vantaa, Finland, Finland version 1.1), in 120 eyes of 60 non-obese individuals in the sitting, supine, and standing positions and again 5 min after standing. They found the IOP in the supine and standing positions to be higher than in the sitting (mean ΔIOP = +1.16 mmHg, *p* < 0.001) and in the standing ones (mean ΔIOP = +1.55 mmHg, *p* < 0.001). This latter difference reduced over time, (mean ΔIOP between supine and standing position 5′ = +0.68 mmHg, *p* < 0.001) [19]. An IOP increase in the supine position was also reported in patients affected by multiple system atrophy (MSA) and Parkinson’s disease (PD) [20]. De Bernardo et al. demonstrated how both healthy subjects and patients with MSA or PD showed IOP increases in the supine position; this increase was higher in patients with MSA [20]. In addition, an IOP increase in obese patients in the supine positions compared to the sitting and the standing positions was firstly reported in 2015 by Geloneck et al. in which, similarly to the present study, the authors measured the IOP of obese subjects in three different positions [21]. The results of the present study not only confirm that ΔIOPs between the supine and other positions were statistically significant (*p* < 0.05) in both obese subjects and non-obese controls but, in addition to previous studies, they showed that ΔIOP in the supine and 5′ standing positions was higher in obese subjects than in non-obese controls (*p* < 0.05).

Several hypotheses have been formulated to explain these findings: the IOP increase in the supine position in non-obese subjects may occur because of choroidal vascular engorgement caused by the redistribution of body fluids in the supine position [22], or an increase in episcleral venous pressure [23]. A higher increase in patients affected by MSA is probably due to direct pressure and volume changes in the vascular compartments within and around the eye, including the periorbital tissues and the intraocular blood volume, most of which lies in the choroid [24]. An IOP increase in obese patients could be explained by the fat mass compression on the abdomen and thorax in supine positions, causing an increase in intra-abdominal and intrathoracic pressure and, thus, elevating the central venous pressure (CVP) [25]. Since the episcleral venous system is a valve-free system, the increase in the CVP is transmitted to the episcleral venous system, reducing the passive outflow of the aqueous humor through the Schlemm’s channel and causing an increase in IOP [23].

In 2017, Lam et al. reconfirmed the Geloneck et al. study [21], and for the first time extended the IOP measurements to obese subjects with a significant reduction in BMI after bariatric surgery [26]. The study showed a non-significant difference in the postural change in IOP between obese subjects and normal-size controls, concluding that obesity was associated just with the increase in IOP in obese patients, but not with the postural changes. The new measurements after the weight loss showed a reduction in IOP that was not statistically significant compared to the values obtained in the same patients in the pre-operative period, showing a weak correlation between the loss of body weight and the reduction in IOP.

Based on these assumptions, we wanted to compare IOP changes, based on postural changes, with the two factors from which the BMI is derived: height and weight. Also in these cases, Figure 1, Figure 2 and Figure 3 and Table 2 showed how both factors are weakly related to variations in IOP with postural changes. Therefore, we cannot consider height and weight as factors directly responsible for the variation of IOP in postural changes. Similarly to Lam et al., our study also shows that the relationship between BMI and the changes in IOP with the postural changes is weak, as shown in Figure 1, Figure 2 and Figure 3 and in Table 2. Among these, the only measurement that differs from the others, with an ρ = 0.432, is the one that relates BMI with the IOP in the supine position 5′. In this case, the positive correlation between the two parameters is a little more evident than the others. On the other hand, contrary to Lam et al., our study found statistically significant differences in the postural change of the IOP between obese subjects and non-obese controls, as will be discussed below.

In addition, our study confirms findings demonstrated by previous studies that there are no significant changes in IOP after a short period of time spent in a supine position. In fact, previous reports have shown how IOP can rise for 30 min after spending time in supine position, while others found no difference between IOP at time 0 and at 15 min [27,28,29].

IOP changes in our study could be due to other factors such as vascular dysfunction secondary to endothelial damage, insulin resistance or autonomic nervous system deficits [30,31,32], resulting overall in a difficult blood flow and in an unstable tissue perfusion. In fact, obesity increases the risk of developing hypertension and atherosclerosis underlying the ischemic hypoperfusion injury [11, 33,34,35].

In 2019, Panon et al. [36] investigated the correlation between the anterior chamber depth (ACD), IOP and BMI, finding a strong positive correlation between ACD and BMI and between IOP and BMI, with a higher IOP in obese subjects compared to non-obese controls, and concluding that the degree of obesity was a significant factor. According to the authors, the reasons for this correlation were due to the large amount of periorbital fat, the increased blood viscosity and the reduced episcleral outflow caused by the leptin-induced oxidative damage that is typically observed in obese subjects [36]. In 2020, Ahn et al. [2], in their study that investigated the relationship between the IOP and obesity on 40,850 patients, also found ocular hypertonus in obese subjects, and traced this condition to increased orbital adipose tissue that increased the episcleral venous pressure, hindering the outflow blood; the increased secretion of cortisol characteristic of obese subjects; and the dysmetabolic syndrome that typically affects such patients [2]. Çekiç et al. [37] investigated the correlation between IOP and the extraocular orbital vessels using ultrasound associated with eco-color Doppler (CDU), and investigated the effects of obesity on retrobulbar flow [11]. This originated from what was argued by Stojanov et al. [38], namely, that the focal accumulation of fat in certain anatomical regions, such as in the retrobulbar region, could cause morphological and functional changes in that body district [38]. Evaluating these parameters in obese and normal-size subjects without vascular diseases and exclusively of white race, because retrobulbar flow is strongly influenced by ethnicity [39], they demonstrated an increase in IOP in obese patients and related decreases in blood-flow velocity in the ophthalmic artery, concluding that the increase in IOP, along with the decrease in retrobulbar blood flow, especially in obese subjects, may increase the risk of developing glaucoma. We must remember that these measurements are only a snapshot of the actual IOP, since it has been shown that IOP has circadian fluctuations that can influence the progression of glaucoma [40,41,42], but this does not invalidate the results of our study where only the pressure differences in different positions, on which circadian variations should have no influence, were evaluated.

For the same reason, the central corneal thickness (CCT) parameter was not included in our analysis. In fact, CCT can influence IOP measurement, with underestimation in thin corneas or overestimation in thick corneas; in addition, the primary outcome of our study was represented by the ΔIOP value. Since CCT does not influence pressure differences in different positions, it was not considered in this study.

The vast majority of the subjects included in the study were of female gender, because it is well known that the number of male subjects undergoing bariatric surgery is a small fraction compared with the number of female patients, despite male obesity rates estimated to be equal to female obesity rates [43]. In fact, considering patients undergoing bariatric surgery, 19.3% were male and 80.7% were female [43]. Since obese subjects were recruited from a bariatric surgery department, the female prevalence of obese subjects’ sample was expected. We also recruited a non-obese subject sample with a similar female/male proportion to have comparable populations. Supplementary Tables S1 and S2 confirm that ΔIOP in the supine positions was greater in obese subjects independently from sex, with a stronger trend in male patients.

A point of strength of this study was the evaluation not only of the IOP measurements of both obese subjects and non-obese controls in different positions, but also the ΔIOP differences in both populations: in this way, it was possible to detect the entity of IOP variation between obese individuals and normal-size controls. In this way, the comparison between populations was not affected by the baseline IOP, and it was possible to investigate more precisely the existence of a greater variability of the IOP in obese subjects than non-obese controls. In fact, as shown in Table 2, statistically significant differences were detected in ΔIOP obtained with the two supine positions and prolonged standing position, with a significant increase in the IOP in obese subjects that was >1.00 mHg in both cases (+1.10 mmHg ΔIOP supine/standing 5′ and +1.02 supine after 5 min (supine 5′)/standing 5′).

It could be considered that a limitation of our study was the use of the TPA instead of Perkins tonometers or Goldmann applanation tonometry (GAT), which is considered the gold standard, but the latter requires a slit lamp, which does not meet the need to measure the ocular tone in various positions. Moreover, obese subjects have extreme difficulty in resting their chin and forehead on the chin guard of the slit lamp to be examined. This is the reason why the study was conducted with the TPA, which uses the same physical principle as GAT to measure IOP and whose reliability is guaranteed by the numerous previous studies that have shown reliable measurements when compared to the GAT in a sitting position [42,44]. It should be taken in account that other external factors could cause IOP variations: for example, emotional changes could play a role, especially in subjects that are undergoing a surgical procedure. In addition, the results of statistical analysis reinforce the evidence for a greater ΔIOP in the supine positions in obese subjects.

## 5. Conclusions

In obese subjects, there was a statistically significant increase in the IOP in the supine positions. BMI is only weakly correlated with IOP and ΔIOP in postural changes. However, further studies that estimate not only the BMI but also evaluate the fat mass distribution with the waist-to-waist ratio, or that distinguish it from the lean mass using bio-impedance analysis, could confirm or eventually deny these findings. In obese subjects, there is an increase in the IOP in the supine position that is greater than in the non-obese population. ΔIOP differences were statistically significant between all the supine positions and the standing 5′ position.

## Figures and Tables

**Table 1:** Summary of IOP measurements in both obese and control subjects in different positions.

Parameter	Standing 5′	Sitting	Supine	Supine 5′	Immediately Standing
	**IOP in different Positions—Obese Subjects**				
Mean ± SD	15.01 ± 2.29 mmHg	15.52 ± 2.84 mmHg	17.13 ± 3.27 mmHg	17.25 ± 2.95 mmHg	15.29 ± 2.38 mmHg
CI 95%	15.01–15.95 mmHg	14.93–16.11 mmHg	16.45–17.81 mmHg	16.64–17.86 mmHg	14.80–15.79 mmHg
Median	15.00 mmHg	15.00 mmHg	16.00 mmHg	16.50 mmHg	15.00 mmHg
Min/Max	11.00/21.00 mmHg	10.00/27.00 mmHg	11.00/26.00 mmHg	13.00/28.00 mmHg	10.00/24.00 mmHg
IQR	3.00 mmHg	3.00 mmHg	4.00 mmHg	4.00 mmHg	2.00 mmHg
KS	0.094	0.002	0.010	0.001	0.039
	**IOP in different Positions—Non obese Controls**				
Mean ± SD	14.88 ± 2.83 mmHg	14.31 ± 2.27 mmHg	15.60 ± 2.90 mmHg	15.81 ± 2.70 mmHg	14.23 ± 2.17 mmHg
CI	14.05/15.70 mmHg	13.65/14.97 mmHg	14.76/16.45 mmHg	15.03/16.60 mmHg	13.60/14.86 mmHg
Median	15.00 mmHg	14.00 mmHg	16.00 mmHg	16.00 mmHg	14.00 mmHg
Min/Max	10.00–21.00 mmHg	9.00–18.00 mmHg	10.00–21.00 mmHg	10.00–20.00 mmHg	9.00–19.00 mmHg
IQR	5.00 mmHg	3.00 mmHg	5.00 mmHg	4.00 mmHg	3.00 mmHg
KS	0.736	0.059	0.370	0.215	0.716

IOP = Intraocular Pressure; SD = Standard Deviation; CI 95% = 95% Confidence Interval; Min/Max error = Minimum and Maximum Error; IQR = Interquartile Range; KS = P Value of Exact Kolmogorov–Smirnov Test; (-) = Data not available.

**Table 2:** Spearman’s rank correlation coefficient (ρ) between Intraocular Pressure measurement obtained in different positions and between Intraocular Pressure Variations trough supine and other positions in obese subjects (*p* value < 0.05).

	**IOP in Different Positions—Obese Subjects**							
Parameter	Standing 5′		Sitting	Supine		Supine 5′		Immediately Standing
**BMI**	0.207		0.231	0.365		0.432		0.316
Height	0.086		0.044	0.051		0.048		0.040
Weight	0.196		0.083	0.291		0.355		0.264
	**ΔIOP in Different Positions—Obese Subjects**							
**Parameter**	ΔIOP Supine/ Standing	ΔIOP Supine/ Standing 5′	ΔIOP Supine/ Sitting	ΔIOP Supine/ Immediately Standing	ΔIOP Supine 5′/ Standing	ΔIOP Supine 5′/ Standing 5′	ΔIOP Supine 5′/ Sitting	ΔIOP Supine 5′/ Immediately Standing
BMI	0.330	0.314	0.325	0.131	0.282	0.311	0.291	0.227
Height	0.076	−0.040	−0.053	−0.046	0.109	−0.012	0.018	0.023
Weight	0.282	0.202	0.195	0.60	0.252	0.208	0.204	0.163

IOP = Intraocular pressure; BMI = Body Mass Index; ΔIOP = Intraocular pressure variations.

**Table 3:** Comparison of intraocular pressure variations detected in different positions both in obese subjects and in non-obese controls.

Parameter	ΔIOP Supine/ Standing 5′	ΔIOP Supine/ Sitting	ΔIOP Supine/ Immediately Standing	ΔIOP Supine 5′/ Standing 5′	ΔIOP Supine 5′/ Sitting	ΔIOP Supine 5′/ Immediately Standing
	**ΔIOP in different Positions—Obese Subjects**					
Mean ± SD	1.83 ± 2.47 mmHg	2.02 ± 2.88 mmHg	2.08 ± 2.55 mmHg	1.96 ± 2.44 mmHg	2.15 ± 2.74 mmHg	2.21 ± 2.17 mmHg
CI 95%	1.12–2.55 mmHg	1.18–2.86 mmHg	1.34–2.82 mmHg	1.25–2.67 mmHg	1.35–2.94 mmHg	1.58–2.84 mmHg
Median	1.50 mmHg	1.50 mmHg	1.50 mmHg	2.00 mmHg	2.00 mmHg	2.00 mmHg
Min/Max	−3.00/9.00 mmHg	−7.00/11.00 mmHg	−2.00/10.00 mmHg	−3.00/8.00 mmHg	−7.00/8.00 mmHg	−1.00/7.00 mmHg
IQR	3.00 mmHg	2.00 mmHg	3.00 mmHg	3.00 mmHg	3.00 mmHg	4.00 mmHg
KS	0.062	0.049	0.068	0.008	0.041	0.101
	**ΔIOP in different Positions—Non-obese Controls**					
Mean ± SD	0.73 ± 1.83 mmHg	1.29 ± 1.54 mmHg	1.38 ± 1.90 mmHg	0.94 ± 1.90 mmHg	1.50 ± 1.64 mmHg	1.58 ± 1.76 mmHg
CI	0.20–1.26 mmHg	0.84–1.74 mmHg	0.82–1.93 mmHg	0.39–1.49 mmHg	1.02–1.98 mmHg	1.07–2.09 mmHg
Median	1.00 mmHg	1.00 mmHg	1.00 mmHg	1.00 mmHg	1.00 mmHg	1.00 mmHg
Min/Max	−3.00/5.00 mmHg	−3.00/5.00 mmHg	−3.00/5.00 mmHg	−3.00/6.00 mmHg	−2.00/4.00 mmHg	−3.00/6.00 mmHg
IQR	3.00 mmHg	2.00 mmHg	2.00 mmHg	2.00 mmHg	3.00 mmHg	3.00 mmHg
KS	0.140	0.093	0.105	0.291	0.146	0.204
ΔIOP Patients – ΔIOP controls	1.10 mmHg	0.73 mmHg	0.70 mmHg	1.02 mmHg	0.65 mmHg	0.63 mmHg
P	0.015	0.272	0.280	0.027	0.336	0.213

ΔIOP = Intraocular pressure variation; SD = Standard Deviation; CI 95% = 95% Confidence Interval; Min/Max error = Minimum and Maximum Error; IQR = Interquartile Range; P Value of Exact Kolmogorov–Smirnov Test; P = level of significance obtained using Mann–Whitney U Test.

## Data Availability

All data generated or analyzed during this study are included in this published article (and its Supplementary Information Files).

## Supplementary Materials

The following supporting information can be downloaded at: https://www.mdpi.com/article/10.3390/jcm12185883/s1, Supplementary Table S1. Comparison of Intraocular Pressure Variations detected in different positions both in female obese subjects and in female non-obese controls. Supplementary Table S2. Comparison of Intraocular Pressure Variations detected in different positions both in male obese subjects and in male non-obese controls.
