# Supplementary material for: Intraocular Pressure Variations in Postural Changes: Comparison between Obese and Non-Obese Controls
Source: J Clin Med. 2023 Sep 10;12(18):5883. doi: 10.3390/jcm12185883 (PMC10531973; doi:10.3390/jcm12185883)
Supplement: Supplementary file 1 [file jcm-12-05883-s001.zip › jcm-2564345-supplementary.pdf]

Supplementary Table S1. Comparison of Intraocular Pressure Variations detected in different positions both in female obese subjects and in female non-obese controls.

Parameter	ΔIOP Supine/ Standing 5'	ΔIOP Supine/ Sitting	ΔIOP Supine/ Immediately Standing	ΔIOP Supine 5'/ Standing 5'	ΔIOP Supine 5'/ Sitting	ΔIOP Supine 5'/ Immediately Standing
**ΔIOP in different Positions – Female Obese Subjects (64)**						
Mean ± SD	1.56±2.24mmHg	1.53±2.20mmHg	1.84±2.60mmHg	1.50±2.14mmHg	1.47±2.71mmHg	1.78±2.08mmHg
CI 95%	1.00-2.12mmHg	0.86-2.20mmHg	1.19-2.49mmHg	0.97-2.03mmHg	0.79-2.15mmHg	1.26-2.30mmHg
Median	1.00mmHg	1.00mmHg	2.00mmHg	2.00mmHg	2.00mmHg	2.00mmHg
Min/Max	-3.00/9.00mmHg	-7.00/10.00mmHg	-2.00/10.00mmHg	-3.00/6.00mmHg	-9.00/8.00mmHg	-1.00/7.00mmHg
IQR	3.00mmHg	3.00mmHg	3.00mmHg	3.00mmHg	3.00mmHg	3.00mmHg
KS	0.243	0.079	0.247	0.138	0.089	0.325
**ΔIOP in different Positions – Female Non-obese Controls (38)**						
Mean ± SD	0.81±1.85mmHg	1.26±1.56mmHg	1.38±2.00mmHg	1.00±1.95mmHg	1.45±1.67mmHg	1.57±1.86mmHg
CI	0.20-1.26mmHg	0.77-1.75mmHg	0.76-2.00mmHg	0.39-1.61mmHg	0.93-1.97mmHg	0.99-2.15mmHg
Median	1.00mmHg	1.00mmHg	1.00mmHg	1.00mmHg	1.00mmHg	1.00mmHg
Min/Max	-3.00/5.00mmHg	-3.00/5.00mmHg	-3.00/5.00mmHg	-3.00/6.00mmHg	-2.00/4.00mmHg	-3.00/6.00mmHg
IQR	3.00mmHg	2.00mmHg	2.00mmHg	2.00mmHg	3.00mmHg	3.00mmHg
KS	0.169	0.166	0.162	0.146	0.119	0.128
ΔIOP Patients – ΔIOP controls	0.75mmHg	0.27mmHg	0.46mmHg	0.50mmHg	0.02mmHg	0.21mmHg
P	**0.043**	0.325	0.279	0.067	0.496	0.314

ΔIOP = Intraocular Pressure Variation; SD = Standard Deviation; CI 95% = 95% Confidence Interval; Min/Max error = Minimum and Maximum Error; IQR = Interquartile Range; P Value of Exact Kolmogorov Smirnov Test; P = level of significance obtained by Mann-Whitney U Test.

Supplementary Table S2. Comparison of Intraocular Pressure Variations detected in different positions both in male obese subjects and in male non-obese controls.

Parameter	ΔIOP Supine/ Standing 5'	ΔIOP Supine/ Sitting	ΔIOP Supine/ Immediately Standing	ΔIOP Supine 5'/ Standing 5'	ΔIOP Supine 5'/ Sitting	ΔIOP Supine 5'/ Immediately Standing
**ΔIOP in different Positions – Male Obese Subjects (28)**						
Mean ± SD	1.86±2.32mmHg	1.79±3.07mmHg	1.82±2.16mmHg	2.39±3.35mmHg	2.32±3.74mmHg	2.36±2.41mmHg
CI 95%	0.96-2.76mmHg	0.59-2.98mmHg	0.98-2.66mmHg	1.09-3.69mmHg	0.87-3.77mmHg	1.42-3.29mmHg
Median	1.00mmHg	1.50mmHg	1.50mmHg	1.50mmHg	2.00mmHg	2.00mmHg
Min/Max	-3.00/7.00mmHg	-3.00/10.00mmHg	-2.00/7.00mmHg	-2.00/8.00mmHg	-4.00/8.00mmHg	-1.00/7.00mmHg
IQR	4.00mmHg	3.00mmHg	3.00mmHg	4.00mmHg	5.00mmHg	4.00mmHg
KS	0.243	0.079	0.247	0.138	0.089	0.325
**ΔIOP in different Positions – Male Non-obese Controls (10)**						
Mean ± SD	-0.10±1.52mmHg	1.30±1.25mmHg	1.30±0.94mmHg	0.00±1.63mmHg	1.40±1.27mmHg	1.40±0.84mmHg
CI	-1.19-0.99mmHg	0.40-2.20mmHg	0.62-1.98mmHg	-1.17-1.17mmHg	0.50-2.30mmHg	0.80-2.00mmHg
Median	-0.50mmHg	1.00mmHg	1.00mmHg	0.00mmHg	1.00mmHg	1.00mmHg
Min/Max	-2.00/2.00mmHg	-1.00/3.00mmHg	0.00/3.00mmHg	-3.00/2.00mmHg	0.00/4.00mmHg	0.00/3.00mmHg
IQR	3.00mmHg	2.00mmHg	1.00mmHg	3.00mmHg	2.00mmHg	1.00mmHg
KS	0.202	0.219	0.245	0.361	0.611	0.523
ΔIOP Patients – ΔIOP controls	1.96mmHg	0.49mmHg	0.52mmHg	2.39mmHg	0.92mmHg	0.96mmHg
P	**0.009**	0.361	0.335	**0.016**	0.231	0.178

ΔIOP = Intraocular Pressure Variation; SD = Standard Deviation; CI 95% = 95% Confidence Interval; Min/Max error = Minimum and Maximum Error; IQR = Interquartile Range; P Value of Exact Kolmogorov Smirnov Test; P = level of significance obtained by Mann-Whitney U Test.
